# The role of salivary galectin-3 and galectin-9 levels in plaque-induced gingivitis and periodontitis

**DOI:** 10.1016/j.heliyon.2023.e19979

**Published:** 2023-09-16

**Authors:** Zerrin Barut, Ahmet Mert Nalbantoğlu, Hilal Korkmaz, Zeynep Demir, Mükerrem Hatipoğlu, Aysun Özkan, Şule Bulut

**Affiliations:** a Department of Biochemistry, Faculty of Dentistry, Antalya Bilim University, Antalya, Turkey; b Department of Periodontology, Faculty of Dentistry, Antalya Bilim University, Antalya, Turkey; c Department of Periodontology, Faculty of Dentistry, Akdeniz University, Antalya, Turkey; d Department of Biology, Faculty of Science, Akdeniz University, Antalya, Turkey; e Department of Periodontology, Faculty of Dentistry, Kyrenia University, Kyrenia, Northern Cyprus, Turkey

**Keywords:** Gingivitis, Periodontitis, Galectin-3, Galectin-9, Saliva

## Abstract

**Background:**

This study aimed to compare the salivary galectin-3 and galectin-9 levels in periodontitis, gingivitis, and periodontally healthy patients.

**Methods:**

This study included 75 non-smokers who were systemically healthy. The clinical periodontal parameters of each participant were recorded. Individuals with periodontal health, gingivitis, and Stage II or Stage III Grade B periodontitis were allocated to the corresponding study groups (n = 25 each). Saliva samples were obtained from all individuals after they abstained from drinking and eating 1 h before sample collection. The galectin-3 and galectin-9 levels in the saliva were analyzed using enzyme-linked immunosorbent assay. One-way analysis of variance, student's t-test, Spearman correlation, and logistic regression were used for statistical analyses.

**Results:**

The galectin-3 and galectin-9 levels were significantly higher in the periodontitis and gingivitis groups than in the healthy group (p < 0.001). The highest galectin-3 and galectin-9 levels were observed in the gingivitis group (p < 0.05). Overall, the galectin-3 levels were significantly higher than the galectin-9 levels in all the groups (p < 0.001).

**Conclusions:**

The salivary galectin-3 and galectin-9 levels were high in patients with periodontitis and gingivitis, suggesting that they could be potential biomarkers for periodontal diseases.

## 1 Introduction

Periodontal diseases are conditions that cause inflammatory changes in the gingival tissues, periodontal ligament (PDL), and hard tissues, including teeth and bones as a result of microbial biofilm which form on the teeth and gingiva. *Porphyromonas gingivalis* and *Aggregatibacter actinomycetemcomitans* are the major pathogens in oral microbiota that have been associated with periodontal diseases [1]. Gingivitis, the most commonly observed type of gingival disease, is associated with dental plaque formation without the loss of clinical attachment. Periodontitis is an inflammatory disease characterized by the presence of inflammation of the supporting tissues of the teeth, resulting in attachment and alveolar bone destruction [2]. Despite extensive hard tissue loss, the teeth can survive and function with advanced restorative procedures [3]; however, the extensive periodontal loss may need tooth extraction. Therefore, periodontal health is crucial for the survival of the teeth.

The host response to the bacterial load in the dental biofilm is one of the major components determining the prognosis of periodontal disease. Host response, which occurs as a response to periodontal infection, comprises the expression of several bioactive agents that are the outcomes of multiple signaling pathways, including growth factors, proinflammatory or anti-inflammatory cytokines, and enzymes [4].

Galectins, a family of mammalian lectins, are crucial in cell homeostasis during the immune response. Extracellular or intracellular galectins play an important role in the differentiation, proliferation, adhesion, and survival of cells as well as in apoptosis and signaling mechanisms [5,6]. Galectin-3 (Gal-3) is released from many cell types including eosinophils, macrophages, monocytes, natural killer cells, dendritic cells, and T or B cells. In addition, some studies have claimed that Gal-3 interacts directly with pathogen-associated molecular pattern [7] receptors and damage-associated molecular pattern receptors [8], which play important roles in host defense against microbes. Structurally different from the other members of the galectin family, galectin-9 (Gal-9) is involved in numerous biological processes, including superoxide production, cell aggregation, chemoattraction, and improvement of cell survival [9].

There are several major inorganic and organic substances in the saliva that act as non-invasive diagnostic materials. Since collecting saliva is an easy, inexpensive, painless, and safe clinical method, it is preferred by both patients and health professionals. Thus, salivary analysis is recommended for the diagnosis and monitoring of many oral and systemic disorders (e.g., cancer, infection, and cardiovascular disorders) [[10], [11], [12]]. Both periodontitis and gingivitis are found moderately associated with cardiovascular, cardiometabolic, and autoimmune diseases and mental ill health [13]. Complicated pathological processes in these disorders may alter saliva -omics that play an important role in the detection of these systemic diseases or local pathologies [14,15]. As a potential diagnostic body fluid, it may provide an evaluation of the pathogenesis and diagnosis of periodontal disease [16]. In dentistry, Gal-3 and Gal-9 were detected in different diseases including periapical granuloma, radicular cyst, or gingival diseases [[17], [18], [19]]. Recent previous studies demonstrated higher Gal-3 levels in serum or gingival crevicular fluid (GCF) of patients with gingivitis or periodontitis [17]. Another study suggested that significant upregulation of Gal-9 mRNA expression was detected in the periodontal pocket of patients with severe periodontal disease [19]. Previous studies analyzed the biomarker in GCF, serum, or periodontal ligament itself [[17], [18], [19]]. Our study aimed to analyze the Gal-3 and Gal-9 levels via saliva, which is a simple and easier collection method. Thus, this study aimed to compare the Gal-3 and Gal-9 levels in the saliva of patients with periodontitis, gingivitis, and periodontal health.

## 2 Material and methods

This cross-sectional study was approved by the Ethics Committee of the Akdeniz University for human research (Protocol number: 70904504/805). The study protocol was performed in accordance with the guidelines outlined in the Helsinki Declaration of 1975 and revised in 2013. The individuals in the study were selected from among the patients who were referred to the university's dental clinic from February 2022 to September 2022. Written informed consent was obtained from all individuals before all procedures. To determine the sample size of the study, a power analysis was performed using G*Power, version 3.1 (Heinrich–Heine–Universität, Düsseldorf, Germany). This was based on the data from a pilot study that had seven samples in each group (total, 21 samples), with a confidence level of 0.05, power of 95%, and effect size of 0.44; thus, the required minimum sample size was 23 specimens in each group. Considering laboratory losses, two samples were added to each group, and the total sample size was 75 (n = 25 in each group).

### 2.1 Selection of the population

This study included 75 non-smokers (50 females and 25 males; age range, 18–67 years) who were systemically healthy. The inclusion criteria were absence of systemic diseases, no ongoing medicinal therapy, no crowns, veneers, or restorations, and presence of more than 20 teeth. The exclusion criteria were presence of a systemic disease, ongoing use of medicines, pregnancy, menstruation, history of periodontal treatment in the last 6 months, or use of any local or systemic antibiotics and anti-inflammatory drugs in the last 2 weeks.

The participants with periodontal health, gingivitis, and periodontitis were categorized into Group H, Group G, and Group P, respectively (n = 25 each). Periodontitis and gingivitis were defined according to the Classification of Periodontal and Peri‐Implant Diseases and Conditions, which is validated in the up-to-date periodontology literature and was revised in the World Workshop in 2017 [2]. Group H participants (5 males and 20 females; mean age, 25.92 years) presented healthy periodontium, no clinical attachment loss (CAL), probing depth (PD) ≤3 mm, and whole-mouth bleeding on probing (BOP) sites <10%. Group G participants (9 males and 16 females; mean age, 27.76 years) presented gingivitis without CAL or alveolar bone loss on radiographs, PD ≤ 3 mm, and whole-mouth BOP sites ≥10%. Group P participants (11 males and 14 females; mean age, 43.88 years) presented Stage II or Stage III Grade B periodontitis including a minimum of two non-adjacent sites with PD 3–4 mm or CAL ≥5 mm and radiographic bone loss of 15–35% or that extending to the middle-third of the root or more. Panoramic radiography was used to analyze the alveolar bone loss. Clinical periodontal parameters of each individual were recorded, including the CAL, PD, and plaque index (PI), which was assessed according to the Silness and Löe PI using a periodontal probe at six sites of the teeth.

### 2.2 Sampling of the saliva

Unstimulated saliva was obtained for this study. The participants were instructed to avoid drinking, eating, or chewing gum 1 h before saliva sampling. Saliva was collected between 09:00 and 11:00 in the morning at a similar room temperature. The unstimulated saliva samples were collected from individuals using a plastic cup and transferred to centrifuge tubes using sterile syringes. The samples were vortexed for 10 min at room temperature to eliminate cells and food debris and to diminish the turbidity of the saliva to protect the accuracy of the analysis [16]. The supernatants were stored at −80 °C until the enzyme-linked immunosorbent assay (ELISA) analysis.

### 2.3 Measurement of galectin-3 and galectin-9 in the saliva samples

The levels of Gal-3 and Gal-9 proteins in the saliva were analyzed using ELISA (Abcam, Discovery Drive, Cambridge Biomedical Campus, Cambridge, UK). Analyses were performed according to the manufacturer's instructions.

A Gal-3 ELISA kit was used to analyze the Gal-3 levels in the saliva. Standard solutions and saliva samples were added to each well, and 50 μL of an antibody cocktail was added to each pre-prepared well. The sealed plates were incubated at room temperature on a plate shaker at 400 rpm for 1 h. After incubation at 37 °C for 1 h, the plate was washed three times using 350 μL of wash buffer. After washing, 100 μL of 3,3′,5,5′-tetramethylbenzidine (TMB) development solution was added to each well and incubated for 10 min in the dark on a plate shaker at 400 rpm. Stop solution (100 μL) was added to each well. An ELISA reader (Thermo-Scientific, 51119200) was used to read the absorbance of all investigated biochemical parameters at 450 nm. The sample concentrations were calculated based on the standard absorbance values.

A Gal-9 ELISA kit was used to analyze the Gal-9 levels in the saliva. The samples and standard solutions were added to each well. After covering the plate, it was incubated for 2 h at 18–25 °C. After incubation at 37 °C for 1 h, the washing procedure was performed thrice with 350 μL of wash buffer. Diluted biotinylated anti-Gal-9 (50 μL) was added to each well. The covered plate was incubated again for 1 h at 18–25 °C. After incubation, the washing process was repeated. Streptavidin-horseradish peroxidase solution (100 μL) was added to each well. The plate was then covered and incubated for 30 min at 18–25 °C. The washing process was repeated after 30 min. Ready-to-use TMB substrate solution (100 μL) was added to each well and incubated for 10–15 min at room temperature in the dark. Additionally, 100 μl of H_2_SO_4_ stop solution was added to all wells. An ELISA reader (Thermo-Scientific, 51119200) was used to read the absorbance of all investigated biochemical parameters at 450 nm. The sample concentrations were calculated based on the standard absorbance values.

### 2.4 Statistical analysis

Statistical analyses were performed using Statistical Product and Service Solutions, version 26.0 (IBM Corp., Armonk, NY, USA). The normality of the data was analyzed using the Kolmogorov–Smirnov test, and the Levene test was performed for homogeneity. The differences between the study groups in the PI, gingival index, and PD were analyzed using one-way analysis of variance and post-hoc Tukey test. Multigroup logistic regression analysis was performed to compare the Gal-3 and Gal-9 levels between the study groups. A correlation analysis was used to examine the relationship between Gal-3 and Gal-9 in the study groups. The level of significance, with a 95% confidence interval, was set at p < 0.05.

## 3 Results

In this study, saliva samples were collected and analyzed from 75 patients with periodontitis, gingivitis, and periodontal health, without any laboratory losses. The limit of detection (LOD) value in our study was 8 pg/mL for Gal-9 and 13.3 pg/mL for Gal-3. There was no sample under these values for Gal-3 and Gal-9. Table 1 shows the periodontal parameters of the study groups. The PI, PD (mm), CAL (mm), and BOP (%) values in Group P were significantly higher than those in Group H and Group G (p < 0.001). Similarly, the periodontal parameters showed significantly higher values in Group G than in Group H (p < 0.001). The salivary levels of Gal-3 and Gal-9 are shown in Table 2. The Gal-3 and Gal-9 levels were significantly higher in Group G and Group P than in Group H (p < 0.001). The Gal-3 and Gal-9 levels were the highest in Group G (p < 0.001). Additionally, the Gal-3 levels were significantly higher than the Gal-9 levels in all the study groups (p < 0.001).

**Table 1:** Saliva levels of Galectin-3 and Galectin-9 (PI: plaque index, PD: probing depth, BOP: bleeding on probing, CAL: clinical atachment level).

Periodontal Parameters	Study Groups			
	Healthy (n = 25)	Gingivitis (n = 25)	Periodontitis (n = 25)	P value
**PI**^a^	0.68 ± 0.02^a,b^	1.19 ± 0.03^a^	1.81 ± 0.03^b^	<0.001
**BOP (%)**	2.13 ± 0.2^a,b^	46.93 ± 5.5^a^	50.87 ± 18.1^b^	<0.001
**CAL (mm)**	0.00 ± 0.002^a,b^	0.58 ± 0.001^a,c^	3.69 ± 0.021^b,c^	<0.001
**PD (mm)**	1.81 ± 0.02^a,b^	2.14 ± 0.02^a^	3.47 ± 0.07^b^	<0.001

Same lowercase in row means statistical difference according to one-way ANOVA post hoc Tukey's HSD (p < 0.05).

a According to the Silness&Löe plaque index.

**Table 2:** Comparison of galectin levels in study groups.

	Saliva Concentration (Mean ± Std)			
	Periodontitis (n = 25)	Gingivitis (n = 25)	Healthy (n = 25)	P value
**Galectin – 3 (ng/μL)**	2.03 ± 0.05^A,a^	2.84 ± 0.056^A,b^	0.67 ± 0.05^A,a,b^	<0.001
**Galectin – 9 (ng/μL)**	0.46 ± 0.02^B,a^	0.66 ± 0.02^B,b^	0.11 ± 0.01^B,a,b^	<0.001
**P value**	<0.001	<0.001	<0.001	
**R value**	0.09	0.15	0.14	

Same lowercase in row means statistical difference according to one-way ANOVA post hoc Tukey's HSD (p < 0.05).

Different uppercase in column means statistical difference according to Student's *t*-test (p < 0.05).

R value of 0.00 indicates no linear relationship, 1.00 indcates a strong linear relationship.

Table 3 shows the bivariate correlation between the Gal-3 and Gal-9 levels in the saliva of all the study groups. Strong positive correlations were observed between Gal-3 and all periodontal parameters (p < 0.001). A strong positive correlation was found between Gal-9 and PD (p < 0.001), without a statistically significant correlation; however, a positive correlation was observed between Gal-9 and PI, BOP, and CAL (p > 0.05).

**Table 3:** Bivariate correlation analysis for saliva Gal-3 and Gal-9 in all patients (PI: plaque index, PD: probing depth, BOP: bleeding on probing, CAL: clinical atachment level).

	Periodontal Parameters							
	PI^a^		BOP (%)		CAL (mm)		PD (mm)	
	R value	P value	R value	P value	R value	P value	R value	P value
**Galectin–3 (ng/μL)**	0.242	**0.036**	0.80	**0.034**	0.43	**0.026**	0.047	**0.002**
**Galectin–9 (ng/μL)**	0.164	0.16	0.20	0.98	0.67	0.87	0.337	**0.003**

Bold P values indicate a significant relationship between periodontal parameters and galectin levels according to Spearman correlation (p < 0.05). R value of 0.00 indicates no linear relationship, 1.00 indcates a strong linear relationship.

a According to the Silness& Löe plaque index.

Prior to regression, the likelihood ratio test was performed in order to reveal the meaningful explanation between the independent and dependent variables. The explanatory power of the model was 86.9% according to Cox&Snell or Nagelkerke R2 values. Group P was 1.637 and 1.047 times more likely to have higher Gal-3 and Gal-9 values than Group H, respectively. Group G was 2.578 and 1.090 times more likely to have higher Gal-3 and Gal-9 values than Group H, respectively (Table 4). Estimation rates of the established model in Group H were 100%, and in Group P and Group G were 92%.

**Table 4:** Multigroup logistic regression analysis.

		B	Standard error	Wald	df	Sig.	Odds Ratio	95% CI for EXP(B)	
								Lower	Upper
**Periodontitis**	**Gal-3**	0.030	14.423	0.020	1.000	0.001	1.637	0.000	1.949
	**Gal-9**	0.086	52.347	12.029	1.000	0.001	1.047	0.000	3.942
	**Constant**	−64.391	16230.250	264.981	1.000	0.001			
**Gingivitis**	**Gal-3**	0.017	20.317	0.000	1.000	0.001	2.578	0.000	2.000
	**Gal-9**	0.046	78.626	0.000	1.000	0.001	1.090	0.000	8.848
	**Constant**	−43.787	16789.494	0.000	1.000	0.001	0.000		
Cox and Snell: 0.869, Nagelkerke: 0.925									

Df: degrees of freedom.

CI: Confidence interval.

## 4 Discussion

The purpose of our study aimed was to analyze the Gal-3 and Gal-9 levels in the saliva samples of patients with periodontitis, gingivitis, and periodontal health. The main result of our study was the increased saliva levels of Gal-3 and Gal-9 in patients with periodontal disease. Periodontal diseases result from a complicated interaction between the dental biofilm located subgingivally and the body's immune response. If dental plaque accumulation increases, the presence of several defense cells in the connective tissues increases, particularly neutrophils, plasma cells, and macrophages, as does the extracellular release of their destructive structures, resulting in inflammation and development of the basic clinical signs of gingivitis and periodontitis.

The most important goals of the novel revised classification of periodontal diseases are early diagnosis, prediction of disease progression, and application of individualized treatment for patients. Although standard clinical and radiographic parameters are important in the diagnosis of periodontal diseases and are useful in detecting evidence of past periodontal disease or confirmation of periodontal health, they do not provide any information on the current disease status or future progression. Many studies have emphasized the importance of diagnostic and prognostic biomarkers [[20], [21], [22], [23], [24]].

A recent review indicated that saliva metabolomics may be a diagnostic tool for various oral diseases including periodontitis and oral cancers [21]. Saliva, which contains many markers (e.g., host cells, proteins, bacteria, and bacterial products), can be easily collected and has been proposed as a diagnostic sample for periodontal disease [16,21,[24], [25]]. Our study revealed that Gal-3 and Gal-9 proteins involved in inflammation increased in the saliva of individuals with periodontitis and gingivitis. Moreover, the salivary Gal-3 and Gal-9 levels were the highest in gingivitis and the lowest in periodontal health.

The activity of neutrophils against microbes is pivotal for an acute response to inflammation. Gal-3 is involved in the different stages of acute inflammation, including neutrophil activation and adhesion, apoptosis mechanism of neutrophils, and degranulation of the mast cells. Additionally, Gal-3 can bind to the integrins on the cell surface and induce inflammation by increasing the adhesion of the neutrophils and vascular endothelial cells.

Gal-3 has several biological functions including cell aggregation, chemoattraction, and apoptosis [26]. Various cells, including monocytes/macrophages, express Gal-9.

There are few studies on Gal-3 and Gal-9 expression in periodontal diseases. A previous study investigated the Gal-3 levels in the gingival crevicular fluid (GCF) in periodontitis, gingivitis, and healthy individuals and found that Gal-3 is a potential biomarker for gingival inflammation; periodontal therapy efficiently reduced the GCF Gal-3 levels [17]. A study comparing the Gal-3 and Gal-9 levels between the control group, radicular cysts group, and periapical granulomas group found that the levels were significantly higher in the periapical granulomas group than in the other groups [18]. The researchers concluded that the increase in these bioactive molecules could be associated with periapical inflammation [17], while another study reported no significant differences in the Gal-3 levels between the radicular cyst, residual radicular cyst, and periapical granuloma cases [27]. Furthermore, Gal-3 expression was found to be higher in the bisphosphonate-associated osteonecrosis of the jaw samples than in the osteoradionecrosis samples [28]. Kasamatsu et al. found higher levels of Gal-3 and significant upregulation of mRNA in the PDL in patients with severe periodontal disease than in healthy individuals. These results suggest that Gal-9 expression is associated with inflammatory responses in the PDL [19].

Gal-3 is known to drive inflammation in various diseases, whereas Gal-9 is known for its immunomodulatory role in various microbial infections. Both are members of the β-galactoside-binding lectin family [29]. Gal-3 is a multifunctional protein that binds proteins in a carbohydrate-dependent and independent manner. It is found predominantly in the cytoplasm and is widely expressed in human tissues, including in all types of immune cells, epithelial cells, and endothelial cells. It is involved in immunity and inflammatory responses and is secreted into the biological fluids, including serum, saliva, and urine. On the other hand, Gal-9 is expressed in all organ systems and is localized in the nucleus, cytoplasm, and extracellular matrix and on the cell surface. Given its role in many different clinical conditions, it is emerging as a new diagnostic and prognostic marker [[29], [30], [31]]. In recent studies, serum and salivary galectin levels were measured using ELISA [31,32], and a significant correlation between the serum and saliva in terms of various biomarker levels was reported [33]. Similarly, there was no significant difference between the saliva and GCF for different galectin levels or the various inflammation markers investigated [34,35]. Salivary Gal-3 levels have been reported to show significant differences in diseases such as heart failure and diabetes. This emphasizes the importance of identifying salivary biomarkers, as Gal-3 and Gal-9 are potential biomarkers for detecting different diseases in their early stages, including pulpal diseases [24,36,37].

To our knowledge, this study is the first to examine the Gal-3 and Gal-9 levels in the saliva of patients with periodontal disease. We found that Gal-3 and Gal-9 proteins are associated with increased inflammation in individuals with periodontitis and gingivitis, and the salivary levels of Gal-3 and Gal-9 were the highest in gingivitis and the lowest in periodontal health. Moreover, when we evaluated the correlation between the clinical parameters and biochemical parameters, there was a positive statistical relationship between Gal-3 and the PI, PD, BOP, and CAL values and a strong positive correlation between Gal-9 and PD. Although there was no statistical correlation between Gal-9 and the other parameters (PI, CAL, and BOP), a positive correlation was reported.

The limitations of our study are that although potential biomarkers for the periodontal disease were investigated in the saliva, changes in the galectin levels after periodontal treatment were not examined. Further studies are warranted to examine the Gal-3 and Gal-9 levels in the GCF or serum. Moreover, a more precise confirmation of the relationship between Gal-3 or Gal-9 levels and periodontitis or gingivitis as well as the post-treatment levels needs to be investigated in further studies. Another limitation of this study is its small sample size. Although the required sample size was calculated using power analysis, a larger sample size is needed to determine a more precise relationship. The limitations of saliva as a diagnostic fluid are the collection method cannot always be standardized, the samples may be contaminated, it cannot be used for the analysis of molecules with large molecular weight, and cannot pass from plasma to saliva. Also, it may cause bias due to factors such as gum, tooth brushing, eating, and drinking. The strength of this study is that it compared the Gal-3 and Gal-9 levels in both periodontitis and gingivitis; therefore, it was possible to compare the increase in the Gal-3 and Gal-9 levels simultaneously in the same sample.

## 5 Conclusion

Within the limitations of this study, increased salivary Gal-3 and Gal-9 levels were found in periodontitis and gingivitis patients. These results suggest that salivary Gal-3 and Gal-9 may be potential biomarkers for periodontal diseases. These molecules can be detected in the saliva of patients with periodontal disease and may be effective in the early diagnosis and treatment of these diseases.

## Author contribution statement

Prof. Şule Bulut: Conceived and designed the experiments; Performed the experiments; Wrote the paper.

Dr. Ahmet Mert Nalbantoğlu: Conceived and designed the experiments; Performed the experiments; Analyzed and interpreted the data; Wrote the paper.

Dr. Zerrin Barut: Conceived and designed the experiments; Performed the experiments; Analyzed and interpreted the data; Contributed reagents, materials, analysis tools or data; Wrote the paper.

Zeynep Demir: Contributed reagents, materials, analysis tools or data.

Dr. Hilal Korkmaz: Contributed reagents, materials, analysis tools or data.

Dr. Mükerrem Hatipoğlu: Contributed reagents, materials, analysis tools or data.

## Data availability statement

The authors do not have permission to share data.

## Declaration of competing interest

The authors declare that they have no known competing financial interests or personal relationships that could have appeared to influence the work reported in this paper.
